# Effects of Expansive Agents on the Early Hydration Kinetics of Cementitious Binders

**DOI:** 10.3390/ma12121900

**Published:** 2019-06-13

**Authors:** Miao Miao, Qingyang Liu, Jian Zhou, Jingjing Feng

**Affiliations:** 1 College of Hydraulic and Civil Engineering, Shandong Agricultural University, Tai’an 271018, China; 2 College of Biology and the Environment, Nanjing Forestry University, Nanjing 210037, China; liuqingyang0807@aliyun.com; 3 College of Materials Science and Engineering, Chongqing University, Chongqing 400045, China; 15213321949@163.com

**Keywords:** expansive agents, early hydration kinetics, cementitious binders, Krstulovic–Dabic model

## Abstract

The addition of expansive agents could overcome the main disadvantages of raw concrete including high brittleness and low tensile strength. Few studies have investigated the early hydration kinetics of expansive cementitious binders, though the findings from the early hydration kinetics are helpful for understanding their technical performances. In this study, mixtures of 3CaO•3Al_2_O_3_•CaSO_4_ and CaSO_4_ (i.e., ZY-type™ expansive agent) with different proportions of mineral admixtures (e.g., fly ash and slag) were added into cement pastes to investigate the early hydration kinetics mechanism of expansive cementitious binders. Early hydration heat evolution rate and cumulative hydration heat were measured by isothermal calorimeter. Kinetic parameters were estimated based on the Krstulovic–Dabic model and Knudsen equations. Mechanical performances of expansive cementitious binders were tested in order to evaluate if they met the basic requirements of shrinkage-compensating materials in technical use. The early hydration heat released from cementitious binders containing ZY-type™ expansive agent was much greater than that released by pure cement, supporting the idea that addition of the expansive agent would improve the reaction of cement. The early hydration kinetic rates were decreased due to the reactions of the mineral admixture (e.g., fly ash or slag) and the ZY-type™ expansive agent in the cement system. The hydration reaction of cementitious binders containing ZY-type™ expansive agent obeyed the Krstulovic–Dabic model well. Three processes are involved in the hydration reaction of cementitious binders containing ZY-type™ expansive agent. These are nucleation and crystal growth (NG), interactions at phase boundaries (I), and diffusion (D). The 14-day expansion rates of cementitious binders containing ZY-type™ expansive agent are in the range of 2.0 × 10^−4^ to 3.5 × 10^−4^, which could meet the basic requirements of anti-cracking performances in technical use according to Chinese industry standard JGJ/T 178-2009. This study could provide an insight into understanding the effects of expansive agents on the hydration and mechanical performances of cementitious binders.

## 1. Introductions

Concrete is a widely-used industrial composite material, which is primarily composed of cement, sand, gravel, water, mineral admixtures (e.g., fly ash, slag, and silica fume), and chemical admixtures [1,2,3]. It is estimated that civil infrastructures including dams, long-span bridges, high-rise buildings, power plants, and harbor constructions could consume more than 10 million cubic meters of mass concrete in China every year [3,4,5]. Yet, the main disadvantage of mass concrete is that it exhibits high brittleness, low tensile strength and high cracking risk caused by concrete shrinkage, which limits its use in large construction projects under extreme environmental conditions [6,7]. The addition of expansive agents in cementitious binders during the industrial process is a practical strategy for enhancing the strength and anti-cracking performance of cement-based materials [8,9]. For instance, expansive agents could compensate for shrinkage of underground concrete structures in order to avoid water leakage [8]. Typical commercial expansive agents are anhydrous calcium sulfoaluminate (3CaO•3Al_2_O_3_•CaSO_4_), calcium oxide (CaO), and magnesia (MgO), and the mixtures of 3CaO•3Al_2_O_3_•CaSO_4_, CaO, and MgO [8,9].

Previous studies indicated that the greater mechanical/anti-cracking performances of cement-based materials are highly dependent on the extent of expansive agent hydration reaction [10,11]. Therefore, understanding the heterogeneous mechanism of cement hydration is necessary for predicting deformations, stress development, and cracking of novel cement-based materials during practical processes for construction projects [12,13]. From a theoretical view, several kinetic equations have been proposed to describe the heterogeneous mechanism of cement early hydration systems. These equations are very important and instructive in understanding interdependencies in studies of composite binders’ early hydration processes [14,15,16]. Though the synthesis of multi-component cement-based materials involves complex hydration kinetics during the whole process, investigations of early hydration processes are of vital importance for selecting appropriate concrete mixtures for real structures of industrial multi-component cement [15,16]. Bernard et al. [17] developed a multiscale micromechanics-hydration model to investigate the hydration reactions of ordinary Portland cement and further study the ageing elasticity of cement-based materials. The Krstulovic–Dabic model has proved to be a robust equation for understanding the effects of additive agents on the early hydration process and mechanism of cementitious materials [16]. Fourmentin et al. [18] concluded that early hydration of cement containing Ca(OH)_2_ was accelerated and exhibited a shorter setting time due to the introduction of calcium oxide. Our previous study indicated that the MgO expansive agent could enhance cement performances in strength development and cracking resistance compared to raw cement [19].

In this study, we applied Krstulovic–Dabic equations to examine the influences of ZY-type™ (i.e., the mixtures of 3CaO•3Al_2_O_3_•CaSO_4_ and CaSO_4_) expansive agent on the early hydration reaction mechanism of cement with or without mineral admixtures. The performances of mechanical properties including deformation and compressive strengths for cementitious materials with the addition of ZY-type™ expansive agent were also examined so that the mechanical properties of expansive cementitious materials could meet the requirement of anti-cracking performances according to the Chinese technical specification for the application of shrinkage-compensating concrete (JGJ/T 178-2009).The results could provide the relevant theoretical and practical basis for the technical use of composite cementitious materials containing ZY-type™ expansive agent during construction projects.

## 2. Materials and Methods

### 2.1. Materials

**Figure 1:**
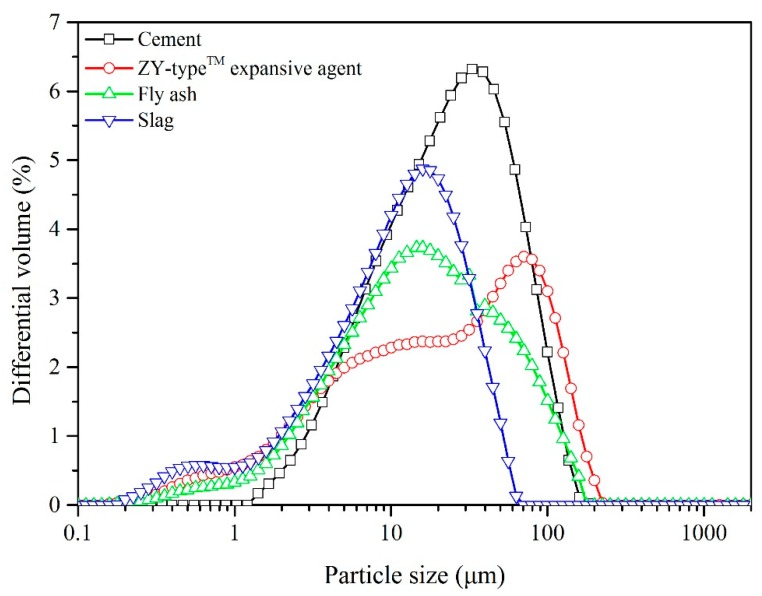
Particle size distributions of cement, slag, ZY-type™ expansive agent, and fly ash. D_50_ values (the particle size corresponding to 50% cumulative particle size distribution): cement, 22.11μm; slag, 11.24 μm; ZY-type™ expansive agent, 22.44 μm; fly ash, 15.89 μm.

Raw Portland cement (PC) obtained from China United Cement Group Co., Ltd. (Beijing, China) was used in this study. Without further purification, this cement conformed to Chinese national standard GB 175-2007 (Table S1). Fly ash (FA) was received from Huaneng Luohuang Power Plant (Chongqing, China) and S95 grade ground granulated blast furnace slag (BS) was purchased from Chongqing Iron & Steel Group Co., Ltd. (Chongqing, China) for hydration kinetics of cementitious binder pastes. The ZY-type™ expansive agent was produced by Chongqing Sansheng Special Building Material Co., Ltd. (Figure S1). The chemical compositions of the cement, fly ash, slag, and ZY-type™ expansive agent are shown in Table 1. The particle size distributions of these raw materials (i.e., cement, fly ash, slag, and ZY-type™ expansive agent) are shown in Figure 1. The water-to-binder ratio (W/B) and proportion of ZY-type™ expansive agent to binder mass were fixed at 0.4 and 6%, respectively. The mix proportions of the six pastes for the hydration kinetics are shown in Table 2.

### 2.2. Test Methods

The early hydration heat evolution rate and the cumulative hydration heat of cement paste systems were measured with an isothermal calorimeter (TAM Air from TA Instruments, Waters, Milford, MA, USA) [19]. The tests were performed at 293 K within 168 h for each tested sample. The raw materials and water were kept at the measured temperature before mixing to avoid temperature differences between the paste and isothermal environment. The pastes were placed into the chamber immediately after manually mixing homogeneously. The early hydration heat emission rate and cumulative hydration heat of the cement paste system were continuously monitored as a function of time. They were measured at the same time, as in the production of modern ready-mixed concrete, the mixture is not cast immediately after the mixing of water and raw materials (as it is transported from the concrete mixing plant to the construction site). Therefore, the heat corresponding with the first peak is never produced inside the cast concrete structures. As a result, in this paper, early hydration kinetics after the end of the induction period are considered [20].

The volume expansions of five mortars containing ZY-type™ expansive agents were determined in the BC-160 Restrained Expansion Measuring Instrument for Mortar (from Beijing Sanyou Instruments Co., Ltd., Beijing, China) with 40 × 40 × 160 mm^3^ molds particularly equipped inward with an I-shaped bracket (composed of a Φ4 vertical steel bar and two brass heads fixed on each bottom of the bar) to simulate the actual structural constraints of steel bar to mortars [19]. Prior to measurement, the mortars were pre-mixed for 4 min. The distance between the two brass heads was measured as the length of the bar after casting. The samples were vibrated for 2 min and then kept in humid air at 293 ± 2 K for ~18–26 h, when the compressive strength of samples was increased to 10 ± 2 MPa, on the basis of the requirements of Chinese national recommendatory standard for expansive agents for concrete (GB/T 23439-2017). After demolding, the initial lengths of the mortar prisms were recorded and then transported into water at 293 K for further measurement. The lengths of the mortar were measured at scheduled times (1, 2, 3, 4, 5, 6, 7, 14, and 28 days) in order to evaluate the expansion performances of tested mortars. The mechanical properties of tested mortars were measured under the same conditions as the volume expansions, except no I-shaped bracket was placed in the mold and the demolding age was 24 h to meet the requirements of GB/T 23439-2017. The compressive strengths of mortars cured in water at 293 K at 3, 7, 28, and 90 days curing were examined. For each test, three repeated tests for one sample were obtained, and the average level was used for comparing the mechanical performances across different kinds of mortars.

### 2.3. Early Hydration Kinetics

The Krstulovic–Dabic kinetics model divides the early hydration of cement into three processes: nucleation and crystal growth (NG), interactions at phase boundaries (I), and diffusion (D) [16,20]. The equation of hydration kinetics for each process is described as follows:

For the nucleation and crystal growth process,

(1) $${\left[-\ln(1-\alpha )\right]}^{1/n}=K_{1}\cdot (t-t_{0})=K_{1}^{\prime }\cdot (t-t_{0});$$

for the phase boundaries process,

(2) $${\left[1-{\left(1-\alpha \right)}^{1/3}\right]}^{1}=K_{2}\cdot R^{-1}\cdot (t-t_{0})=K_{2}^{\prime }\cdot (t-t_{0});$$

for the diffusion process,

(3) $${\left[1-{\left(1-\alpha \right)}^{1/3}\right]}^{2}=K_{3}\cdot R^{-2}\cdot (t-t_{0})=K_{3}^{\prime }\cdot (t-t_{0})$$

where *α* is the degree of hydration, which is defined as the fraction of reacted cement; *K*_1_, *K*_2_, and *K*_3_ describe the rate constant for NG, I, and D, respectively; *R* stands for the radius of the reacting particle; *K*_1_′, *K*_2_′, and *K*_3_′ indicate the apparent rate constant for NG, I, and D, respectively, and *n* shows the extent of geometrical crystal growth and its value is usually is in the range of 1 to 3 (n = 1 for needles, n = 2 for sheets and n = 3 for isotropic growth) [20].

In the Krstulovic–Dabic model, the NG process dominated for *α* < *α_NG_* and *t* < *t_NG_*, and the equation could be transformed into the differential form (Equation (4)). The same process is true for interactions at phase boundaries (Equation (5)) and diffusion processes (Equation (6)) [20]. The differential equation of hydration kinetics for each process is

(4) $$d\alpha /dt=F_{1}(\alpha )=K_{1}^{\prime }\cdot n\cdot (1-\alpha )\cdot {\left[-\ln(1-\alpha )\right]}^{(n-1)/n}$$

(5) $$d\alpha /dt=F_{2}(\alpha )=K_{2}^{\prime }\cdot 3{\left(1-\alpha \right)}^{2/3}$$

(6) $$d\alpha /dt=F_{3}(\alpha )=K_{3}^{\prime }\cdot 3{\left(1-\alpha \right)}^{2/3}/\left[2-{\left(2-\alpha \right)}^{1/3}\right].$$

The main parameters of hydration kinetics, including rate constant, apparent rate constant, and extents of geometrical crystal growth could be calculated according to Equations (4)–(6) if the degree of hydration reaction, *α*, could be determined.

The variable, *α*, as a function of hydration time, can be represented as the ratio of the cumulative hydration heat at time, *t*, to the ultimate total hydration heat, *Q_max_*, in Equation (7).

(7) $$\alpha (t)=Q(t)/Q_{\max}$$

A formula of hydration kinetics proposed by Knudsen (Equation (8)) [21] used to obtain the ultimate total hydration heat, *Q_max_*:

(8) $$\frac{1}{Q(t)}=\frac{1}{Q_{\max}}+\frac{t_{50}}{Q_{\max}(t-t_{0})}$$

In the Knudsen equation, *Q* describes the released heat measured by isothermal calorimeter as a function of hydration time, *t*_0_ is hydration time at the end of induction period, *t* − *t*_0_ is the hydration time starting from the acceleration period, and *t*_50_ is the hydration time when the cumulative hydration heat is 50% of the total hydration heat [16].

The hydration exothermic curves of the composite cementitious binders obtained were the comprehensive manifestation of the early hydration reaction of cement, with a faster reaction rate, and the pozzolanic reaction of mineral admixtures and the hydration reaction of the expansive agent, both with slower rates [19]. The Krstulovic–Dabic model is based on the early hydration exothermic data only and presents an overall apparent hydration process of cement-based materials [20]. The kinetic parameters for each process (i.e., NG, I, and D) could not be compared across different kinds of cement-based materials systems because these reactions occur at the same time and are affected by each other. Consequently, the early hydration mechanism for each cement-based material could be interpreted as a homogeneous body with the same hydration activity [16].

## 3. Results and Discussion

### 3.1. Characteristics of Early Hydration Heat of Composite Binders

**Figure 2:**
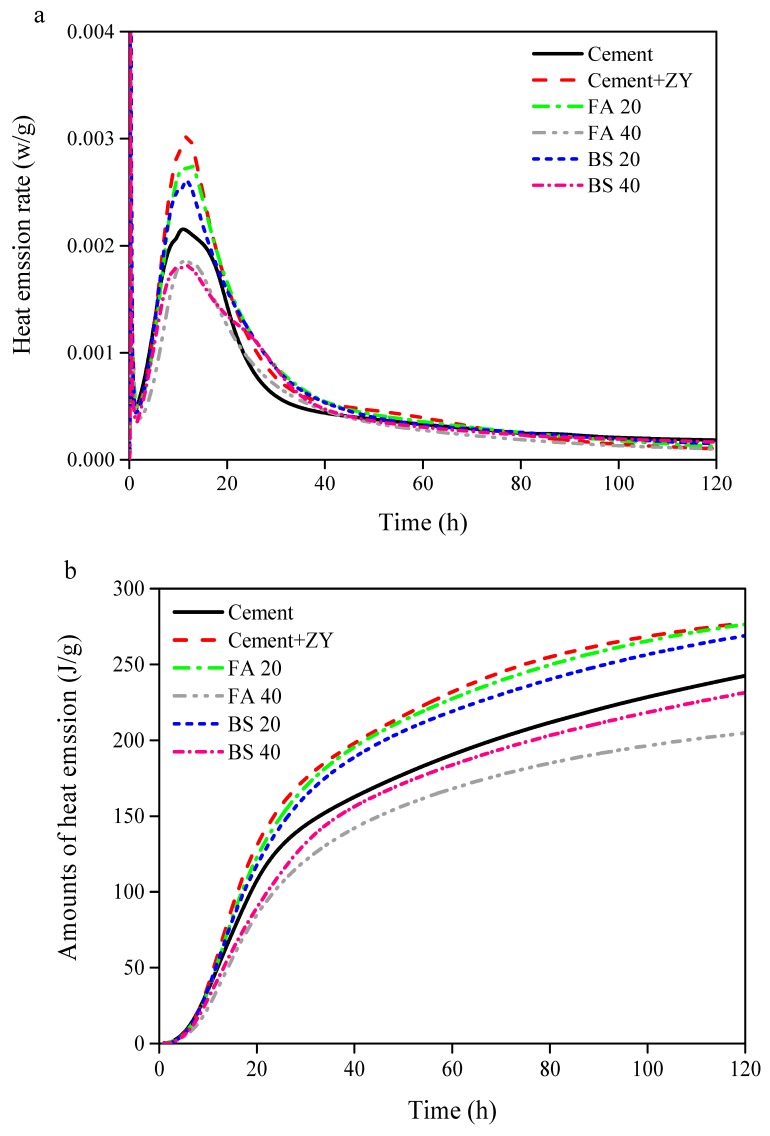
(**a**) Early hydration heat emission rate of cement pastes at 293 K and (**b**) early hydration heat of cement pastes at 293 K. The detailed compositions of the six cement pastes are shown in Table 2.

The early hydration heat evolution rates and cumulative hydration heats of the cement–expansive agent–mineral admixture systems in the proportions of 20%–40% were tested, and the influence of the various compositions on the heat of the hydration were analyzed (Figure 2). After adding expansive agents to the cement (Figure 2a), the induction period of expansive binder material ended earlier (~1.8 h) than pure cement (~2 h) because the expansive agent could accelerate hydration of cement clinkers (i.e., C_3_S and C_2_S) by consumption of the cement hydration product Ca(OH)_2_ in its own reaction with water [22]. The lower second peak of heat evolution rates was observed later (~12 h) than that of cement (~10 h), indicating that the reactivity of ZY-type™ expansive agent was lower than that of cement. With the addition of reactive slag (i.e., BS 20, 20% wt. and BS 40, 40% wt.) or lower reactivity fly ash (i.e., FA 20, 20% wt. and FA 40, 40% wt.) in cement containing expansive agents as expansive cementitious binders, the second peak of heat evolution rates declined by ~6%–45% compared to that of cement. This resulted from the combined effects of the expansive agent and additive (i.e., slag and fly ash) in the cement system.

As seen in Figure 2b, the early hydration of cement paste with ZY-type™ expansive agent released much higher heat than that of cement, proving additions of ZY-type™ expansive agent would improve the cement reaction [23]. Because the fly ash or slag could weaken the hydration-promoting effect of ZY-type™ expansive agent due to their reactions with Ca(OH)_2_ [24], the heat emissions released from FA 20 and BS 20 were still higher than from cement paste without expansive agent. Lower heat emissions were observed in FA 40 and BS 40 than in FA 20 and BS 20, resulting from the higher proportions of slag and fly ash (40% versus 20%). With the increase of mineral admixture content, the cumulative hydration heat of each paste decreased significantly. The higher the content, the more the hydration heat decreased, and the reduction was not proportional to the content. When the slag content was 20% and 40%, the cumulative hydration heats at 120 h were 260 and 225 J, which were lower by 2.9% and 16.2% respectively, than those of cement paste with expansive agent. When the fly ash and slag content were both 40%, the cumulative hydration heat of 120 h was lower by 26.0% and 16.2% respectively than that of cement paste with expansive agent; the addition of fly ash caused a larger reduction than slag as it had lower reactivity due to the lower amount of amorphous SiO_2_ or Al_2_O_3_ in fly ash compared to that in slag [25].

### 3.2. Analysis of Early Hydration Kinetic Parameters

The hydration rate curve (dα/dt) and the simulated curves including F_1_(α), F_2_(α), and F_3_(α) were estimated according to the Knudsen equation, using heat emissions data measured by an isothermal calorimeter. Figure S2 shows the hydration rate curves of blended pastes estimated at 293 K. As seen from Figure S2, the hydration process of cement and cement containing ZY-type™ expansive agent involve three processes, i.e., NG, D, and I. Furthermore, F_1_(α), F_2_(α), and F_3_(α) could simulate the hydration rate curve of cement paste well, which proves the hydration reaction of the cement-ZY-type™ expansive agent system were controlled by a multiple reaction mechanism.

Table 3 lists the main parameters of the three processes of early hydration kinetics, calculated by the Krstulovic–Dabic model. It can be observed from Table 3 that at the hydration temperature of 293 K, the n value was relatively lower for the cement pastes with the ZY-type™ expansive agent than without the expansive agent. When mineral admixture (fly ash or slag) was added into the paste with ZY-type™ expansive agent, the n value gradually decreased. Adding the same amount of fly ash and slag (i.e., FA 20 versus BS 20; FA 40 versus BS 40), the n values of pastes containing slag was always smaller than that of pastes containing fly ash (FA 20, 1.792 versus BS 20 1.788, FA 40, 1.741 versus BS 40, 1.749), which indicated that the effects of slag on crystal growth were greater than those of fly ash in the expansive paste.

With the addition of expansive agent, the apparent reaction rate constant of the NG process K_1_′ decreased by 7.7%, and with the increase of mineral admixture, K_1_′ decreased further in the range of 7.7%–26.4% (Table 3). With the replacement of cement with 20% fly ash or slag with cement paste incorporating ZY-type™ expansive agent, K_1_′ decreased by 7.7% and 10.5%, respectively, while with 40% fly ash or slag, K_1_′ decreased by 14.4% and 26.4%, respectively (Table 3).

The variation in apparent reaction rate constants of I and D processes, K_2_′ and K_3_′, was similar to that of K_1_′ (Figure S2). However, the specific values of the three apparent reaction rate constants exhibited quite different trends. The apparent reaction rate constant of the NG process (K_1_′) was about 3–5 times higher than that of the I process (K_2_′) and 20 times higher than that of the D process (K_3_′). The early hydration reaction of components in the expansive binder system in the NG process was an autocatalytic reaction, and the hydration product formed at a high rate more easily. As the hydration reaction progressed, Ca(OH)_2_ in the liquid phase was saturated to form a relatively strong alkaline solution, eroding the vitreous phase of fly ash and slag in a pozzolanic reaction [22]. In D process, the hydration reaction and the pozzolanic reaction made the porosity and permeability of the pastes reduce significantly. The Ca(OH)_2_ crystals and unhydrated particles in the pastes were covered by the C–S–H layer with little permeability [6,18], so it was difficult for the water in the system to approach the unreacted particles, which increased diffusion resistance, and led to the slowing of the reaction rate of D process [24].

The apparent reaction rate constants of the I process (K_2_′) for BS 20 (n = 0.00955) and BS 40 (n = 0.00715) were smaller than those of the I process (K_2_′) for FA 20 (n = 0.0105) and FA 40 (n = 0.00961), indicating that the I process was significantly affected by slag due to its higher reactivity compared to fly ash (Table 3). The reaction rates of the D process across four materials (i.e., FA 20, FA 40, BS 20, and BS 40) were slower than those of cement with the expansive agent because the extra consumption of hexagonal plates of Ca(OH)_2_ would have led to finer pore and denser microstructure, resulting in the lower hydration kinetic rate of D process [22].

### 3.3. Mechanical Properties and Deformation of Mortars

With the addition of the expansive agent, the three-day compressive strength (35.2 MPa) of mortar containing ZY-type™ expansive agent was larger than that of pure cement (28.5 MPa) (Table 4). This could be attributed to the expansive agent delaying the hydration of the cement causing less expansion and stiffness to occur before the mixtures hardened. Similarly, 7, 28, and 90 day compressive strengths of mortar with ZY-type™ expansive agent were higher than those of pure cement, supporting that idea that adding ZY-type™ expansive agent could enhance the mechanical performances of pure cement. When 20% or 40% of the cement was replaced with fly ash or slag, the compressive strengths of composite binder mortars were all lower than those of pure cement mortar. This was associated with their lower reactivity and the competitive consumption of Ca(OH)_2_ by the expansive agent, weakening the major roles of the expansive agent in the enhancement of mechanical properties in the cement system [8]. The compressive strengths of mortars across four materials (FA 20, FA 40, BS 20, and BS 40) decreased by 1.8%–26.8% in comparison to the pure cement.

**Figure 3:**
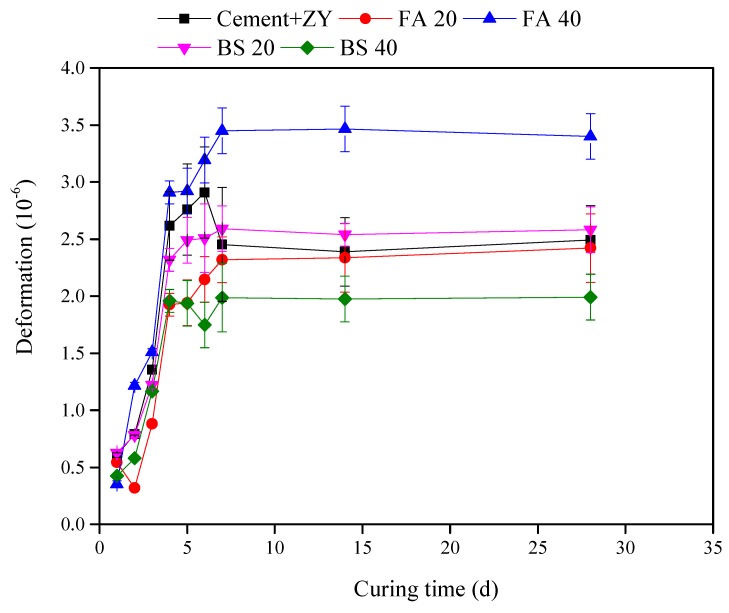
Free expansion rate of mortars cured in water at 293 K.

With the addition of 6% ZY-type™ expansive agent, the expansion rates of five mortars containing expansive agent increased rapidly in the first 7 days from 0.5 × 10^−4^ to 3.5 × 10^−4^, and then remained stable (Figure 3). The 14 day expansion rates of five materials including cement with ZY, FA 20, FA 40, BS 20, and BS 40 ranged from 2.0 × 10^−4^ to 3.5 × 10^−4^, which was higher than the lowest requirement for mortars (1.5 × 10^−4^) in anti-cracking performances in the Chinese technical specification for application of shrinkage-compensating concrete (JGJ/T 178-2009) [26].

### 3.4. Comparisons with Other Expansive Cementitious Binders

Table 5 summarizes several recent studies on the hydration reactions of binders with expansive agents. Cao et al. [19] and Winnefeld et al. [27] did not investigate the effects of expansive agents on early hydration kinetics of binders, though their studies utilized multiple methods to determine hydration processes. Wang et al. [28] illustrated the effects of sewage sludge on the early hydration kinetics of binders. However, sewage sludge is not the kind of commercial expansive agent that could be used, as the hydration reaction of binders containing sewage sludge (NG and D) is different to the hydration reaction of binders containing expansive agents, which involves three processes (NG, I, and D). Currently, ZY-type™ expansive agent is widely used for compensating shrinkage in China. Our study provides experimental data for filling the gaps between the theoretical and practical bases for the technical use of cement with ZY-type™ expansive agent. To the best of our knowledge, our study is the first to provide the details of early hydration reaction on binders containing the ZY-type™ expansive agent. Future studies may focus on hydration kinetics using multiple models that may be more generalizable to complex binder systems [17,29].

## 4. Conclusions

This study investigated the influences of ZY-type™ expansive agent on the early hydration kinetics of cement with mineral admixtures (fly ash or slag). The mechanical performances of cement mixed with ZY-type™ expansive agent were measured to evaluate if this kind of material could meet the technical requirements of the Chinese technical specification for anti-cracking performances of cement-based materials. The cement with ZY-type™ expansive agent was the best fit in the Krstulovic–Dabic model. The early hydration kinetics of different cementitious binders incorporating ZY-type™ expansive agent experienced three processes, NG, I, and D. The reactions of ZY-type™ expansive agent and mineral admixture in binder systems decrease the hydration kinetic parameters (e.g., apparent reaction rate constants). The resulting materials could meet the technical requirements of the Chinese technical specification for the anti-cracking performance of shrinkage-compensating concrete. Our study provides, for the first time, an experimental investigation to evaluate the role of ZY-type™ expansive agent in the early hydration kinetics of composite cementitious systems.

## Figures and Tables

**Table 1:** Chemical compositions of cement, fly ash, slag, and ZY-type™ expansive agent (wt.%).

Materials	SiO_2_	Al_2_O_3_	Fe_2_O_3_	CaO	MgO	SO_3_	Na_2_O_e__q_	*f*-CaO	LOI
Cement	21.62	4.35	3.45	64.40	3.45	2.25	0.50	0.90	1.25
Fly ash	46.89	24.53	13.55	1.35	2.07	0.63	1.57	-	5.68
Slag	32.70	14.03	0.50	39.00	8.99	0.20	0.52	-	0.78
ZY	7.24	13.66	2.23	32.13	2.50	33.71	0.12	-	6.83

Note: Na_2_O_eq_ = Na_2_O + 0.658K_2_O, *f*-CaO = free CaO, and LOI = loss on ignition.

**Table 2:** Mix proportions of pastes (wt.%).

Sample	W/B	Cement	Fly Ash	Slag	ZY-type™ Expansive Agent
Cement	0.4	100	0	0	0
Cement + ZY	0.4	94	0	0	6
FA20	0.4	74	20	0	6
FA40	0.4	54	40	0	6
BS20	0.4	74	0	20	6
BS40	0.4	54	0	40	6

Note: W/B = water to binder ratio.

**Table 3:** Main parameters of early hydration kinetics of blended pastes.

Samples	n	K_1_′	K_2_′	K_3_′	Kinetics Mechanism
Cement	1.946	0.0489	0.0133	0.00269	NG-I-D
Cement + ZY	1.818	0.0451	0.0121	0.00266	NG-I-D
FA 20	1.792	0.0417	0.0105	0.00216	NG-I-D
FA 40	1.741	0.0386	0.00961	0.00201	NG-I-D
BS 20	1.788	0.0404	0.00955	0.00191	NG-I-D
BS 40	1.649	0.0332	0.00715	0.00147	NG-I-D

**Table 4:** Compressive strength (MPa) of mortars cured at 293 K with different scheduled periods.

Samples	3 d	7 d	28 d	90 d
Cement	28.5	37.7	54.2	64.9
Cement + ZY	35.2	42.2	59.8	69.0
FA 20	23.7	34.6	50.4	58.2
FA 40	21.1	28.2	39.7	51.7
BS 20	25.9	35.3	53.9	63.7
BS 40	25.4	32.2	49.9	59.3

**Table 5:** Overview of early hydration kinetics of selected expansive cementitious binders.

Study	Expansive Agent	Early Hydration Kinetics	Technical Use *
Cao et al. [19]	MgO	n.a.	Yes
Winnefeld et al. [27]	CaSO_4_	n.a.	Yes
Wang et al. [28]	Sewage sludge	NG-D	Yes
Our study	ZY-type™	NG-I-D	Yes

n.a. = not available; * The lowest requirement of expansion rates in anti-cracking performance for technical use of concrete is 1.5 × 10^−4^, according to Chinese industry standard JGJ/T 178-2009.

## Supplementary Materials

The following are available online at https://www.mdpi.com/1996-1944/12/12/1900/s1, Figure S1. X-ray diffraction (XRD) spectrum for ZY-type™ expansive agent; Figure S2. Early hydration rate curves of cement blends at 293 K. a. Cement; b. Cement + ZY; c. FA 20; d. FA 40; e. BS 20; f. BS 40; Table S1. The physical properties of raw cement (from China United Cement Group Co., Ltd.) conformed to Chinese national standard for common Portland cement (GB 175-2007).
