# Supplementary material for: Effects of Expansive Agents on the Early Hydration Kinetics of Cementitious Binders
Source: Materials (Basel). 2019 Jun 13;12(12):1900. doi: 10.3390/ma12121900 (PMC6631045; doi:10.3390/ma12121900)
Supplement: Supplementary file 1 [file materials-12-01900-s001.pdf]

Supplementary Materials

Effects of Expansive Agents on the Early Hydration Kinetics of Cementitious Binders

Miao Miao [1,3,*], Qingyang Liu [2], Jian Zhou [3] and Jingjing Feng [1,*]

[1] College of Hydraulic and Civil Engineering, Shandong Agricultural University, Tai'an 271018, China
[2] College of Biology and the Environment, Nanjing Forestry University, Nanjing 210037, China; liuqingyang0807@aliyun.com
[3] College of Materials Science and Engineering, Chongqing University, Chongqing 400045, China; 15213321949@163.com
* Correspondence: miao_thu@163.com (M.M.); jingjing_Feng@163.com (J.F.)

Table S1. The physical properties of raw cement (from China United Cement Group Co., Ltd.) conformed to Chinese national standard for common Portland cement (GB 175-2007)

Material	Specific surface area(m^2/kg)	Density(g/cm^3)	Water demand(%)	Initial setting time(min)	Final setting time (min)	Stability
Cement	350	3.15	25.6	132	198	qualified

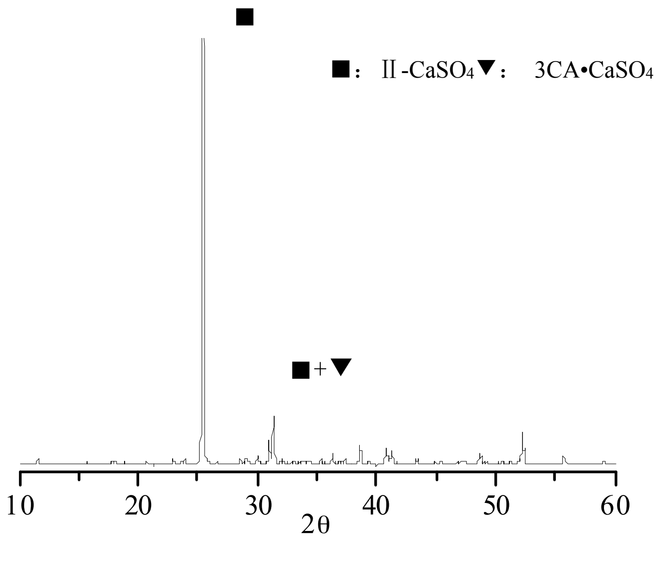

Figure S1. X-ray diffraction (XRD) spectrum for ZY-type™ expansive agent.

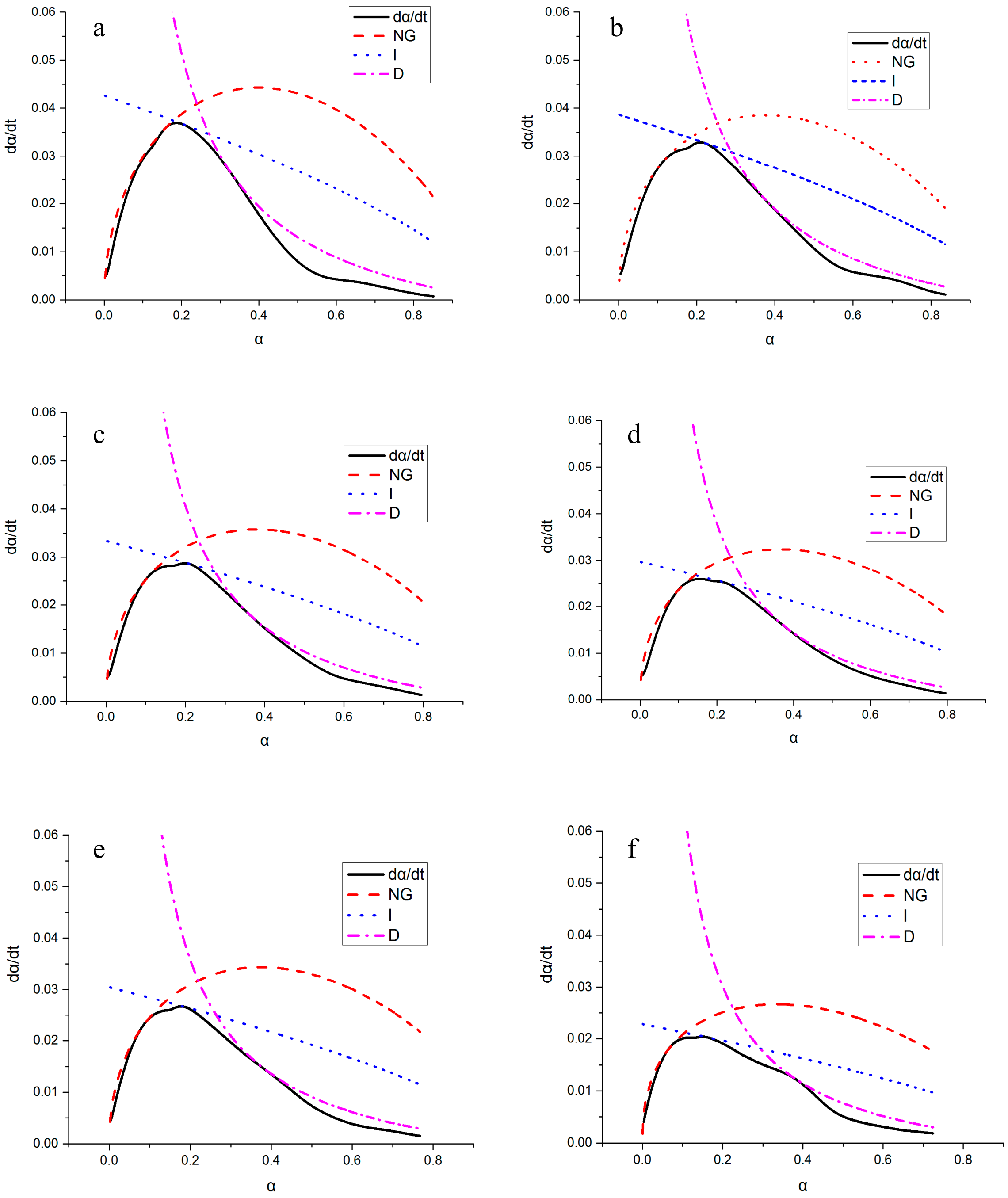

Figure S2. Early hydration rate curves of cement blends at 293 K. a. Cement; b. Cement+ ZY; c. FA 20; d. FA 40; e. BS 20; f. BS 40.
